# Anchote (*Coccinia abyssinica*) starch extraction, characterization and bioethanol generation from its pulp/waste

**DOI:** 10.1016/j.heliyon.2023.e14320

**Published:** 2023-03-06

**Authors:** Etalema Desta Tulu, Ramesh Duraisamy, Belay Haile Kebede, Alemu Mekonnen Tura

**Affiliations:** Arba Minch University, College of Natural and Computational Sciences, Chemistry Department, P.O. Box 21, Arba Minch, Ethiopia

**Keywords:** Anchote, Pulp/wastes, Starch, Bio-ethanol, Fermentation, Distillation

## Abstract

A polysaccharide molecule called starch exists in nature and is cheap, renewable, biodegradable, and readily accessible. The main objective of this project is to extract and characterize anchote (*Coccinia abyssinica*) starch, as well as to generate bio-ethanol from its pulp/waste. The anchote sample used in this investigation came from Ethiopia’s western Oromia region. Anchote (*Coccinia abyssinica*) starch was extracted, and the pulp from the tuber was peeled and the supernatant isolated for bio-ethanol production. The extracted starch from anchote and bio-ethanol from pulps were characterized by physicochemical, functional properties and FT-IR analysis. The result of physicochemical and functional properties of anchote starch was found to be pH (4.44), WHC (112%), Solubility (5.03%), swelling power (5.781%), Gelatinization temperature (53.33 °C), WAC (2 g/g), bulk density (0.605 g/cm^3^) and OAC (3 g/g). According to this FTIR research, anchote starch has a chemical structure that is similar to corn, cassava, and potato starch. As the results showing a promising alcoholic content (25% v/v) and the existence of bioethanol being validated by a combustion test, the pulps/wastes recovered from anchote are also good sources of bioethanol. Finally, the FTIR spectroscopic analysis revealed that ethyl alcohol was produced from anchote pulps/waste after acid hydrolysis, fermentation, and distillation.

## 1 Introduction

**Fig. 1:**
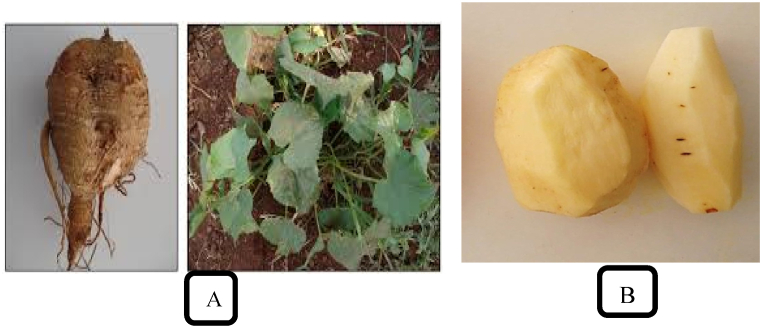
(A) Anchote (*Coccinia abyssinica*) tuber and plant [1] (B) white color of anchote after peeling (Captured by Etalema Desta). (For interpretation of the references to color in this figure legend, the reader is referred to the Web version of this article.)

One of the significant root crops grown in Ethiopia’s west, south, and southwest is anchote (*Coccinia abyssinica*; Fig. 1A and B). It is a subsistence crop that is frequently grown to cover gaps in food security during the months of famine (June to September). Anchote is a sufficient source of protein, carbohydrates, calcium, iron, and zinc. In medicine, it is used to treat gonorrhea, tuberculosis, cancer, malaria, and other conditions. Due to the highest output from small scale farming, it also generates economic incomes because it is grown as a cash crop. The importance of anchote extends to social importance (served only during special ceremonies like thanks giving day, weddings, betrothal, circumcision, birthdays, and “Meskel holiday”) [1]. High quality root crop anchote offers a viable alternative for extraction of starch.

A polysaccharide molecule called starch exists in nature and is cheap, renewable, biodegradable, and readily accessible. In order to provide a method for storing carbohydrates, the plastids of plant tissues produce starch, a water-soluble polyglucan. Since the beginning of civilization, starch derived from plant storage organs has most likely been the major calorie source in the human diet. Humans have maintained their reliance on high-yielding domesticated starch-forming crops such as grains to meet food demands, animal production, and many non-food purposes since the beginning of agriculture and the development of complex societies [2]. The qualities of a crop’s starch determine its suitability for processing and the final product’s quality. Gelatinization, viscosity, tackiness, retrogradation inclination, granule size distribution, and non-starch components are all important characteristics. In the granules, the ratio of amylose to amylopectin, as well as their shape, determine the starch characteristics [2,3]. In various cereals, waxy and non-waxy genotypes, as well as sweet genotypes, provide starch with variable flavors and processes. The starch content of root and tuber crops can be very high, and their output per growing area can be higher than cereals. Root crops are a good way to meet a growing population’s calorie needs. This sector is particularly promising for the production and processing of tropical root crops like cassava, sweet potato, and yams [3]. There are many plants that can be utilized to make starch. Along with some underutilized legumes (sword bean, jack bean), legumes (lima beans, garbanzo beans, lentil beans, red kidney beans, navy beans, faba beans, mung beans, adzuki beans, field pea, cowpea, beach pea, green pea, and groundnut) are also included in the list of grains: wheat, corn, rice, barley, oat, sorghum, millet, and rye (banana, plantain, mango, and pawpaw). According to Alcázar-Alay and Meireles [4], starch granules can be found in seeds, tubers, roots, stems, leaves, fruits, and pollen. The need to promote anchote as a novel starch source is crucial given the expanding need for starches in the food industry.

Leftover pulps after the extraction of starch from anchote should contribute significantly to environmental contamination. Converting the pulp/waste to bio-ethanol reduce the negative impact to the environment. Bioethanol is a fermented alcohol made through microbial fermentation. However, despite environmental concerns, the manufacture of bioethanol from biomass may ultimately eliminate the need for crude oil. Because of its high octane rating and high vaporization heat, bioethanol can be used as a mixed fuel in gasoline engines [5]. Bio-ethanol can be made from a range of biomass sources, including, but not limited to, agricultural and forestry byproducts. Biomass feedstock can be categorized into a wide number of groups depending on the chemical composition of the raw material, such as whether it contains elements that go into making cellulose, sugar, or starch. To make the most of the feedstock, future bio refinery techniques should combine the synthesis of bioethanol with that of additional value-added chemical compounds and biofuels [6]. Historically utilized as an alternative fuel, bioethanol is the most popular biofuel used in transportation. In 1984, the first countries to use bioethanol as a fuel for internal combustion engines (ICEs) were Germany and France. Bioethanol has been consumed in Brazil since 1925. Bioethanol was widely utilized in Europe and the US up to the early 1900s [7].

Generally speaking, the feedstock pulp can be used to create an eco-friendly bio engine or bioethanol (as a byproduct). Compared to gasoline, bioethanol has a wider range of flammability restrictions, faster flame speeds, and greater vaporization temperatures. These characteristics allow for a higher compression ratio, a quicker burn period, and a leaner burn engine in an internal combustion engine, all of which theoretically result in advantages over gasoline in terms of efficiency. Fermentable sugars can be made from basic materials that contain sugar, but glucose needs to be made by hydrolyzing starchy plants. Pretreatment of lignocellulosic biomass is necessary to make the cellulose structures available to enzymes before hydrolysis [8,9]. The main objective of the study was extraction of starch from Anchote (*Coccinia abyssinica*) and Production of bio-ethanol from co-generated waste during the extraction of starch from anchote.

## 2 Materials and methods

### 2.1 Sample collection and preparation

**Fig. 2:**
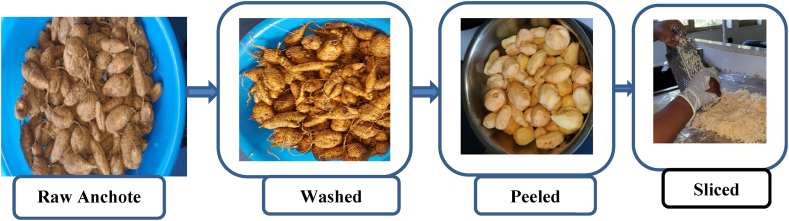
Sample preparation of Raw Anchote (captured by Etalema Desta).

10 kg of white matured anchote roots were purchased from the local market of Nekemte, Ethiopia. The sample was preserved in polyethylene bags and transported to Arba Minch University’s advanced chemistry laboratory. The sample was preserved at room temperature for further use. To eliminate foreign contaminants from the batch, the anchote tubers underwent a comprehensive sorting process. The Anchote tubers that were determined to be healthy and suitable for testing were cleaned and washed to get rid of any adhering dirt, soil, and extraneous contaminants. With a Jagson slicer, the tubers were peeled and cut into chips that were 2–2.5 cm thick, as seen in Fig. 2 below.

### 2.2 Extraction of starch from anchote

Fresh anchote roots were cleaned, peeled, and cut into cubes about 1 cm in size before being ground in a high-speed lab scale blender (BOXIYA, Model- HJ-767/GS-PBJ-01, Germany). After stirring the pulp in 10 times its amount of water for 5 min, it was filtered through two layers of cheesecloth. The top liquid was decanted and discarded after the filtrate was left to stand for 2 h to allow the starch to settle. The suspension was passed through a double-fold cheese cloth filter, and the filtrate was held for 12 h to sediment. The resulting sediment was washed twice with distilled water after the supernatant was discarded. The finished sediment was dried in a drying oven for 24 h at 45 °C. The dried starch was grounded and passed through 250 μm and kept in air plastic containers for experimental analysis [[10], [11], [12]]. The pulp peeled from anchote tuber and supernatant isolated was reserved for bio-ethanol production. The pulp/waste was dried for a week in sun and grinded by using Laboratory grinder (BOXIYA, Model- HJ-767/GS-PBJ-01, Germany).

### 2.3 Preparation of bio-ethanol

#### 2.3.1 Saccharification of anchote pulp/waste

100 g of grinded pulp obtained from dried pulp/waste of anchote was mixed with 1 L of water (as 1:10 w/w ratio) and was taken into water bath. Saccharification/acid hydrolysis (using 50 mL of 5 M HCl) at 85 °C was carried out about 1 h by continuous stirring with the help of magnetic stirrer in order. After saccharification the treated mash was cooled and under taken for filtration process to isolate the desired material for fermentation [5,13].

#### 2.3.2 Inoculums preparation and activation

Each 1000 mL laboratory fermenter (flask) held 1000 mL of substrate and was autoclave sterilized at 121 °C for 20 min. The substrates' fermentation media’s pH was set to 4.5 and maintained at 30 °C. The substrate was sterilized for 24 h in an incubator cabinet [7]. In the current work, anchote pulps were fermented to produce bioethanol using Saccharomyces cerevisiae yeast (SCY). In order to have hygienic and antibacterial benefits, the yeast was activated. Each inoculum propagation container was filled with urea, di-ammonium phosphate (DAP), and salt to provide nutrients and encourage the growth of microorganisms. The injected microorganisms were then further stimulated in a water bath with a mechanical shaker at a controlled temperature (at 30 °C).

#### 2.3.3 Fermentation and distillation

**Fig. 3:**
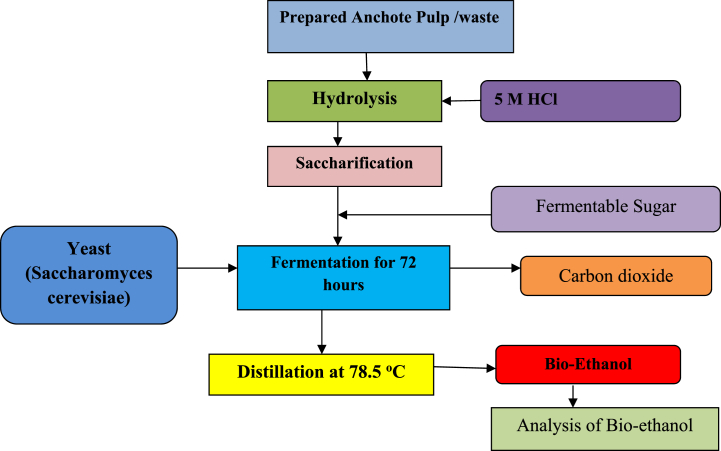
Flow diagram for the production of bioethanol from anchote pulps [9].

In a laminar flow cabinet, bio-ethanol fermentation was carried out in an Erlenmeyer flask as a fermenter. The fermentation was carried out on acid hydrolysates derived from substrate hydrolysis. The samples were then fermented for 72 h (at pH 5.5) at a constant temperature of 30 °C to finish the fermentation [8]. The fermenting sample was collected and distilled using laboratory simple/fractional distillation equipment. Separately, the sample was heated to 78.5 °C in a distillation flask. The specific gravity method [9] was used to determine the volume of distilled wash (bioethanol) production (yield). The process flow diagram for the production of bioethanol is shown in Fig. 3.

### 2.4 Sample characterization

#### 2.4.1 Physico-chemical analysis of anchote starch

##### 2.4.1.1 Measurement of pH

3 mL of distilled water were combined with 1 g of starch. 20 mL of distilled water were added to the mixture to make the slurry. The pH of the starch was determined using a Jenway digital pH meter model 1100 [14].

##### 2.4.1.2 Moisture content determination

Petri plate was dried for 1 h at 100°5 °C, cooled in a desiccator for 30 min, then weighed (w_1_). 5 g of the anchote starch were placed into a dry, pre-weighed Petri dish to determine its weight (w_2_). A container containing a sample was heated to 100 °C in a vacuum oven. The sample was dried for 3 h, put into a desiccator, cooled for 30 min, and weighed once again (w3). According to the literature, the following equation was used to determine the percentage of moisture content [14]. as:

(1) $$\%\;moisture=\frac{w_{2}-w_{3}}{w_{2}-w_{1}}\times 100$$

Total Solid (%) = 100 − % moisture (w/w)

where,

*w*_*1*_ - Weight of dried empty container (g), *w*_2_-weight of container with sample before drying (g)
*w*_*2*_ - *w*_*1*_ is weight of sample (g), *w*_3_-weight of container with sample after drying (g), *w*_2_ – *w*_3_ is weight loss (g)

##### 2.4.1.3 Structural analysis of starch by FT-IR

When the material was thoroughly ground with appropriately dried pure KBr (1:100 w/w), pellets were created and examined by compression. The spectra were acquired in absorbance mode with a resolution of 4 cm^−1^ from 4000 to 400 cm^−1^. Prior to scanning each sample, a pure KBr background value was collected [15].

##### 2.4.1.4 Stability

An aqueous starch suspension with a 1% starch concentration was created by boiling 0.2 g of starch in 20 mL of water for an hour at 90 °C while stirring the water bath. The starch paste was allowed to cool to room temperature before use. A spectrophotometer was used to measure the absorbance at 640 nm while the starch paste was kept at 4 °C (SPECORD 50 plus, Analytikjena, Germany). By taking readings every 24 h for seven days, the pastes' stability was confirmed [15].

##### 2.4.1.5 Turbidity

A 1% aqueous starch suspension was cooked for 1 h at 90 °C in a water bath while being constantly stirred. At 30 °C, the starch paste was chilled for an hour. The samples were stored at 4 °C for five days, and turbidity was measured using a spectrophotometer every day against a water blank (SPECORD 50 plus, Analytikjena, Germany) [14].

#### 2.4.2 Functional properties analysis of anchote starch

##### 2.4.2.1 Water binding capacity (WBC)

15 mL of distilled water were used to dissolve 5 g of starch. The mixture was centrifuged for 10 min at 3000 rpm after 1 h of stirring. The supernatant was drained after being separated from the wet starch and weighed [15].

(2) % WBC = (Bound water)/(Weight of sample) × 100

##### 2.4.2.2 Swelling and solubility

5 g of starch was dissolved in 20 mL of distilled water, placed in pre-weighed 50 mL centrifuge tubes, and shaken at 60, 70, 80, and 90 °C for 30 min. The suspension was centrifuged at 3500 rpm for 10 min. The supernatant was gathered in a Petri plate and dried at 103 °C for 12 h. After decantation, the weight of the larger granules was estimated. The swelling power and percent solubility were calculated using the following formulas [16]:

(3) $$Swelling\;power=\frac{Weight\;of\;Swollen\;Granules}{Weight\;of\;Sample-weight\;of\;dissolved\;starch}\times 100$$

(4) $$Solubility\;\left(\%\right)=\frac{weight\;of\;dried\;starch\;in\;petri\;dish}{Sample\;weight}\times 100$$

##### 2.4.2.3 Gelatinization temperature

1 g of anchote starch and 10 mL of distilled water were added to a beaker, and the mixture was agitated to create homogenized slurry. In a hot water bath, the sample beaker was inserted and agitated continually until it gelled. According to Onwuka’s approach [17], the temperature was measured with a thermometer 30 s after gelation.

##### 2.4.2.4 Water absorption capacity (WAC)

The water absorption capacity was determined using Onwuka’s technique. 1 g of Anchote starch was weighed and put into a weighed graduated conical centrifuge tube. The sample was mixed with a Waring spin mixer, 10 mL of distilled water was added, and the mixture was left at room temperature for 30 min. The mixture was centrifuged at 3500 rpm for 30 min. Using a 10 mL measuring cylinder, the free water (supernatant) volume was determined. The amount of water absorbed was determined by multiplying the total amount of water by the free water amount by 1 g/mL [17].

##### 2.4.2.5 Bulk density (BD)

The sample was properly filed into a graduated measuring cylinder with a capacity of 10 mL, and the cylinder was weighed. The bottom of the cylinder was lightly tapped on the laboratory bench many times after filing to the 10 mL mark until the sample level did not decline. According to Onwuka [18], bulk density (g/mL) was calculated by dividing the sample’s weight (g) by its volume (mL).

##### 2.4.2.6 Oil absorption capacity (OAC)

2 g of flour sample was mixed for 30 s at 200 rpm with 12.5 mL of distilled water. After complete dispersion, 12.5 mL of vegetable oil was progressively added, and the mixture was mixed for an additional 30 s. A centrifuge tube containing the mixed sample was filled, and it was spun for 5 min at 1600 rpm. The amount of oil that had separated from the sample after centrifugation was measured directly from the tube. The amount of oil that could be maintained in suspension in 1 g of sample served as the benchmark for emulsion capacity [19].

#### 2.4.3 Analysis of bioethanol

##### 2.4.3.1 Density

Emtron’s technique was used in this case. According to the expression [7], the density of the produced bioethanol was calculated by dividing the volume of the bioethanol distillate from each fermentative organism by the original mass of the sample used.

(5) $$Density\;\left(g/{cm}^{3}\right)=\frac{mass\;of\;sample\;\left(g\right)}{Volume\;of\;ethanol\;produced\;\left({cm}^{3}\right)}$$

##### 2.4.3.2 Qualitative test for ethanol

Two drops of chromic acid were introduced to a test tube containing two drops of acetone and four drops of distilled bioethanol. The mixture was vigorously shaken. The presence of ethanol is confirmed by the change in color of the mixture, which forms blue-green precipitates within seconds after adding drops of chromic acid [6].

##### 2.4.3.3 Identification of bio-ethanol by color

In a test tube, 5 mL of distilled fermented wash/bio-ethanol sample was mixed to 0.5 g potassium dichromate and 1 mL of H_2_SO_4_. The sample solution’s color was noticed [7].

##### 2.4.3.4 FTIR analysis

FTIR was used to analyze the composition and purity of the distilled fermented ethanol produced from anchote pulp/waste. A Spectrum 65 FTIR (Perkin Elmer, Germany) was used to work in the 4000-400 cm^−1^ wavenumber region using NaCl Windows at room temperature. Using the proper FTIR software, the materials' data and scanned spectra were obtained [9].

##### 2.4.3.5 Quantitative estimation of ethanol

The weight of distilled water was weighed first, followed by the weight of dry and empty specific gravity bottles. The weight of the bottles was measured after the specific gravity bottles were dried and filled with the study’s produced fermented alcohol. Using these data, the specific gravity of the ethanol was calculated, and the amount of alcohol in fermented distillate was calculated using the AOAC table [6].

## 3 Results and discussions

### 3.1 Physico-chemical and functional properties of anchote starch

The results of physicochemical and functional properties of starch isolated from anchote (*Coccinia abyssinica*) are presented in Table 1.

**Table 1:** The Physico-chemical and Functional properties of Starch Extracted from Anchote.

S.No	Parameters	Present Study (mean ± SD)	Comparison with previously reported	References
1	Yield (%)	70.41±0.35	78.71 ± 0.07	[9]
2	pH	4.44 ± 0.01	4.48 ± 0.03	[10]
3	Moisture Content	11.5 ± 0.5	9.36 ± 2.12	[10]
4	Water holding Capacity (%)	112 ± 4.53	107	[10]
5	Solubility (%)	5.03 ± 0.03	6.66	[10]
6	Swelling power (%)	5.781 ± 0.24	5.06	[20]
7	Stability	1.348 ± 0.01	–	–
8	Turbidity	1.134 ± 0.02	–	–
9	Gelatinization temperature (°C)	53.33 ± 1.00	62.0 ± 1.72	[21]
10	Water Absorption Capacity (g/g)	2.00 ± 0.00	1.08	[21]
11	Bulk Density (g/cm^3^)	0.605 ± 0.01	0.59–0.68	[21]
12	Oil Absorption capacity (g/g)	3.00 ± 0.012	2.42–3.35	[19]

According to Table 1, the yield of anchote starch as a percentage was 70.41 ± 0.35%. The production of anchote starch here is less than it was in the earlier [10] report. According to Zhu [22], the amount of starch that can be extracted from anchote depends on the extraction technique utilized and the size of the anchote plantation. Extraction rates range from 80% to 11%. The drying conditions of the raw materials, the timing of harvest and root storage, the health of the roots utilized for extraction, and the environmental conditions for producing the crop are just a few artificial elements that might affect the starch production [22]. The pH of anchote starch was discovered to be 4.44, indicating strong acidity, which was supported by the low pH value. This value falls between the published ranges for fermented (pH = 3.78) and native (pH = 6.68) anchote starch. According to prior research by Pamella Fronza et al. [23], carboxyl groups, which are functional groups of amino acids found in proteins, may be responsible for the acidity of starch.

The moisture level of the starches used in the current study was 11.5 ± 0.5%, which was within the safe range for starch storage without lowering the starch’s quality. The moisture content calculates the samples' dry matter and water contents indirectly [24]. Any sample with a moisture content of less than 14% can prevent microbial development and, as a result, maintain storage stability, according to research by Ojo et al. [25]. As a result, the starch sample’s moisture level, which ranges from 0 to 12%, is appropriate for efficient starch storage without the risk of microbial contamination. As stated by Eze [24] low moisture content resulted in the manufacture of a product that was more shelf stable. Research shows that samples with moisture levels above 14% are not stable at room temperature and, in essence, allow existing organisms to begin multiplying, producing tastes and unpleasant aromas.

The swelling power and solubility of anchote starch were measured after heating at 60 °C, and the results showed that they were 5.781 and 5.03%, respectively. Both values show a breakdown in intermolecular interaction, allowing hydrogen bonds to form stronger interactions with water molecules as previously reported by Sit et al. [11]. The capacity of starch granules to swell is directly connected to the amounts of amylose and amylopectin present, and these components frequently undergo structural changes when heated. The phenomenon of starch solubility and swelling power is a component of the gelatinization process. As a result, the process’s operating temperature will depend on the kind of starch being examined. The hydrogen bonds that cause the granules to swell and expose the hydroxyls of the starch chains and favor the production of long-chain amylopectin of the starch chains are broken during the gelatinization process, allowing water molecules to bind to the accessible hydroxyl groups and causing the granules to swell. Moreover, at temperatures when the majority of granules are gelatinized, a higher solubility of starch has been connected to the same physicochemical mechanism [11,20,23].

At 60 °C, anchote starch was shown to have a 112% water-holding capacity. Anchote starch’s water-binding capability was identical to the previously reported finding (Table 1). Temperature has a direct relationship with solubility, capacity for swelling, and capacity to store water. The source, the swelling ability, the inter-association forces in the amorphous and crystalline domain, and the presence of other compounds are variables that can impact starch’s solubility. One crucial physicochemical factor that affects the functional qualities of starch is solubility [11]. The variance in WBC among various starches may have been influenced by the degree to which water binding sites were available differently in each starch [11]. This outcome might be brought about by the starch hydroxyl group’s propensity to create covalent and hydrogen bonds with starch chains rather than water [[10], [21]].

The starch pastes' stability was tested constantly for seven days, every 24 h, and the average result was 1.348. The starch pastes' stability improved after the first day, remained stable until the fifth day, and then began to deteriorate. This demonstrates that the starch extracted was stable. The cause for the initial increase in stability could be the settling of bigger molecules or contaminants in the starch paste, which increases the stability [12]. The turbidity of the suspensions was measured by comparing gelatinized starch suspensions from anchote samples to water, with the average result shown in Table 1. All starch solutions gradually became more turbid throughout the course of the five days that the starch gels were kept at 4 °C. According to Ashri et al. [14], a variety of variables have been associated to turbidity in starches during storage, including granule enlargement, granule remnants, leached amylose and amylopectin, amylose and amylopectin chain length, intra or interbonding, lipid and cross-linking substitution.

The water absorption capacity (WAC) of anchote starches was 2 g/g, according to Table 1. After being completely hydrated, the starch granules were capable of absorbing twice as much water as they weighed. The amount of water that the granules will absorb when starch is dissolved in an excessive amount of water without being boiled depends on their water absorption capacity. As a result, the granule’s solvent volume increases while it still maintains its granular structure. The ratio and composition of amylose and amylopectin, as well as the surface characteristics of the starch granules, are the key variables that influence the WAC. The WAC of anchote starch was substantially greater than that of potato starch (0.67 g/g) according to Hellemans et al. [26]. The improved handling, enhanced moistness, and softer texture of baked items could all stem from the increased water binding capacity of these starches, which could also lessen dough stickiness. Anchote starch’s oil absorption capacity was reported to be 3 g/g. Food products' ability to absorb oil improves mouth feel and starch retention, making it an important feature in food compositions [18].

The bulk density of the current study was 0.605 g/cm^3^, as shown in Table 1. For technological applications, starch density is significant. This measure represents the content of a material per unit of real volume occupied by the material. According to Moorthy [20], cassava starch has a bulk density of 0.59–0.68 g/cm^3^. Bulk density values can help with packing, handling, and processing. Instant commodities necessitate lower bulk densities. The temperatures at which starch isolated from anchote sample gelatinizes were determined to be 53.33 °C (Table 1). Overall, the starch sample’s gelatinization temperature was lower than previously reported. Alterations in amylose quantity, shape, distribution, and the capacity of starch granules to bind water may be impacted by different gelatinization temperatures [26]. Certain foods that are heat-labile at high temperatures may benefit from the lower gelatinization temperatures of starch samples. For low-temperature food processing, such as batter coating and processed meat products, low-gelatinization temperature flours and starches may be helpful. Cooking is facilitated by low gelatinization temperatures, and cooking time and heat can be lowered to improve food processing efficiency [27].

### 3.2 Structural analysis of anchote starch

**Fig. 4:**
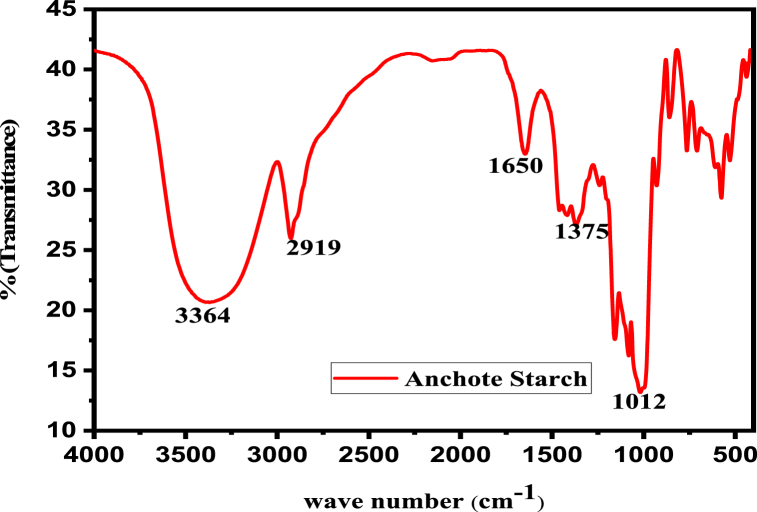
FT-IR spectrum of Starch Extracted from Anchote tuber.

Fourier Transform Infrared Red (FT-IR) spectroscopy was used to identify the functional groups of many compounds in the plant [28]. Fig. 4 depicts the results of an FT-IR study of starch extracted from anchote. The results were interpreted based on the appearance of various bands on the FT-IR spectra that corresponded to specific types of functional groups. The discovered individual bands were assigned to a single functional category based on their wave numbers.

This spectrum can be split into five sections for simpler reading. There are five parts of the mid-infrared light spectrum (4000–400 cm^−1^). The five spectra had absorption bands at 3364, 2919, 1650, 1375, and 1012 cm^−1^, which, respectively, show that starch has an OH, C–H, O–C–O, C–H symmetric bending, and C–O stretching functional group. With wave numbers ranging from 3520 to 3217 cm^−1^, the results show a broad and strong absorption band corresponding to the OH group strains. The band’s amplitude reveals the existence of intermolecular hydrogen bonds. The extension vibrations C–H have wave numbers between 2919 and 2930 cm^−1^. The band at 2925 cm^−1^ is caused by the stretching vibration of methyl C–H. At 1650 cm^−1^ absorption peak would be produced by the OH group’s C–O bending. It was also possible to deduce the existence of C–H symmetrical scissoring of the CH_2_OH molecule using the absorbance peak at 1375 cm^−1^ [27]. Due to strain deformations brought on by C–O stretching, polysaccharide wave numbers range from 1060 to 990 cm^−1^. The outcomes showed the molecules of amylose and amylopectin starch. Numerous remarkable absorbancies are caused by the anti-symmetric stretching vibration of C–O, C–C, and C–O–C at 1156 cm^−1^ and 1080 cm^−1^. According to this FTIR investigation, the chemical structure of anchote starch is the same as that of corn, cassava, and potato starch [28].

### 3.3 Bio ethanol analysis

After separating starch, the synthesis of ethanol from anchote pulps/waste was explored. Fermentation was performed on all parts in order to produce ethanol. The cellulosic material was fermented into bioethanol in the experimental vessel containing anchote pulps. Alcoholometry was used to determine the alcoholic content of the prepared bioethanol after distillation, and it was found to be 25% (v/v).

#### 3.3.1 Qualitative analysis of bioethanol using dichromate test

**Fig. 5:**
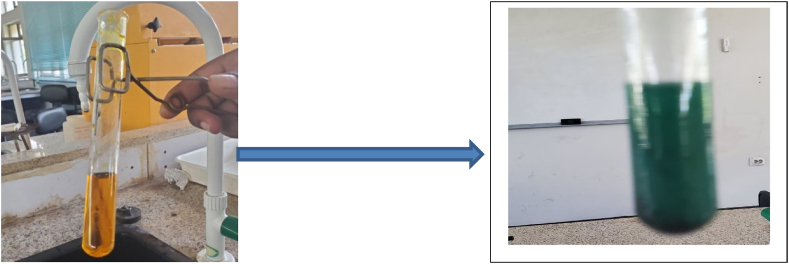
Confirmatory test of Ethanol.

As 1 mL of 2% potassium dichromate solution was added to 5 mL of purified bioethanol, the yellowish orange color of the dichromate turned green, indicating the presence of ethyl alcohol in the sample. Clearly, anchote pulp or waste was fermented to generate the product. The current study uses a combustion test to further validate the presence of ethyl alcohol in the analyzed materials [21]. The sample vapor that had been heated and boiled entirely lit during this test, producing a light blue flame. This study confirmed the existence of bioethanol once more (Fig. 5).

#### 3.3.2 Determining specific gravity of bioethanol

The present investigation’s findings show that the fermented ethanol with anchote pulp/waste that was hydrolyzed by 5% (v/v) hydrochloric acid has a specific gravity of 0.801 g/cm3. In general, it can be said that the hydrolyzed substrate made up of anchote pulp/waste and HCl has shown greater conversion efficiency because its specific gravity is considerably closer to 0.78.

#### 3.3.3 FT-IR spectra of bio-ethanol

**Fig. 6:**
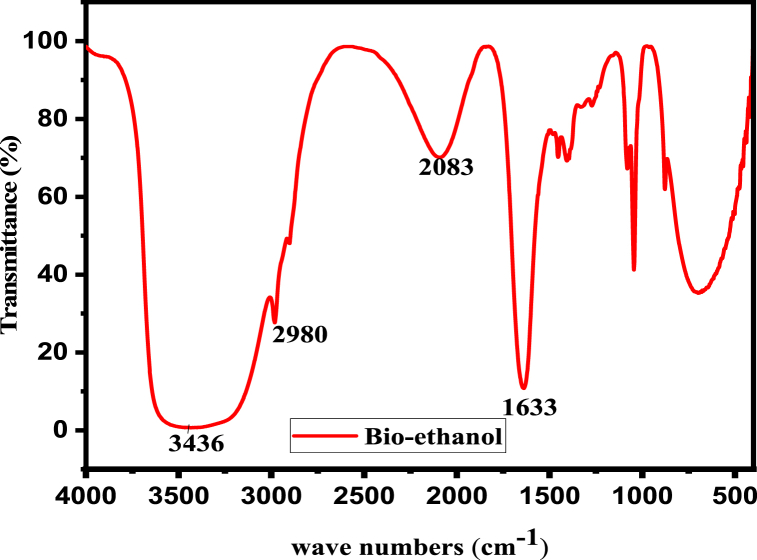
FTIR spectra of distilled bioethanol made from anchote pulp/waste.

FT-IR spectroscopy was used to evaluate bio-ethanol generated from anchote pulps/waste between 4000 and 400 cm^−1^. As shown in Fig. 6, the transmission spectra were acquired using NaCl pellets as the transparent window. The results were analyzed based on the appearance of various bands on the FT-IR spectra. The discovered individual bands were assigned to a single functional category based on their wave numbers.

The FT-IR characterization of distilled bioethanol (25%) was performed, and Fig. 6 shows the peak picking spectrum. The stretching vibrations of the O–H and C–H in alcohols cause unique IR absorptions. In the 3700-3000 cm^−1^ region, the analyzed bioethanol displays a strong broad band at 3436 cm^−1^, showing that alcohols are stretching O–H. The vibrations of C–H stretching are confirmed by the bands at 2980 cm^−1^. The symmetric stretching modes of the –CH_2_ and –CH_3_ groups were attributed to the bands between 3000 and 2840 cm^-^1 (C–H stretching occurs at 2980 cm^−1^) [6]. Additionally, the peak at 1633 cm^−1^ denotes absorbed water and is a typical stretching vibration of the H–O–H in water molecules. The infrared spectrum reached a peak at 2083 cm^−1^. This FTIR spectroscopy analysis revealed that after acid hydrolysis, fermentation, and distillation, the product recovered from anchote pulps/waste was bio-ethanol.

## 4 Conclusion

This research focuses on the extraction and characterization of the physicochemical, functional, and structural properties of anchote (*Coccinia abyssinica*) starch as well as the production of bioethanol from anchote pulps and wastes. When compared to previously published literature, the outcome of physicochemical attributes of anchote was found to be satisfactory. According to this FTIR research, anchote starch has a chemical structure that is similar to corn, cassava, and potato starch. The results show a promising alcoholic content (25% v/v), and a combustion test that confirms the presence of bioethanol also shows that the pulps and wastes recovered from anchote are excellent sources of the fuel. In general, the FTIR spectroscopic examination came to the conclusion that the end product of acid hydrolysis, fermentation, and distillation of anchote pulps and waste was bio-ethanol.

### 4.1 Limitation of the study

Further optimization study must be done on future for production bioethanol from the anchote pulps/wastes as well as for waste water treatment from sludge left behind after hydrolysis is done.

## Declarations

### Author contribution

Etalema Desta Tulu: Conceived and designed the experiments; Contributed reagents, materials, analysis tools or data; Performed the experiments; Analyzed and interpreted the data; Wrote the Paper

Ramesh Duraisamy, Belay Haile Kebede, Alemu Mekonnen Tura: Conceived and designed the experiments; Performed the experiments; Analyzed and interpreted the data; Wrote the Paper

### Funding statement

This research did not receive any specific grant from funding agencies in the public, commercial, or not-for-profit sectors.

### Data availability statement

Data included in article/supplementary material/referenced in article.

### Declaration of interest’s statement

The authors declare no conflict of interest.
